# Shift in precipitation regime promotes interspecific hybridization of introduced *Coffea* species

**DOI:** 10.1002/ece3.2055

**Published:** 2016-04-08

**Authors:** Céline Gomez, Marc Despinoy, Serge Hamon, Perla Hamon, Danyela Salmon, Doffou Sélastique Akaffou, Hyacinthe Legnate, Alexandre de Kochko, Morgan Mangeas, Valérie Poncet

**Affiliations:** ^1^ IRDUMR DIADEBP 6450134394Montpellier Cedex 5France; ^2^ IRDUMR ESPACE DEV (S140)BP A598848Cedex NouméaNouvelle Calédonie; ^3^ DDRBP 238698846Nouméa CedexNouvelle Calédonie; ^4^ Université J. Lorougnon GuédéB150DaloaCôte d'Ivoire; ^5^ CNRA01 BP 1740Abidjan 01Côte d'Ivoire

**Keywords:** Adaptation, bioclimatic envelope, climate change, *Coffea*, flowering phenology, hybrid zone, introduction, New Caledonia, niche, precipitation

## Abstract

The frequency of plant species introductions has increased in a highly connected world, modifying species distribution patterns to include areas outside their natural ranges. These introductions provide the opportunity to gain new insight into the importance of flowering phenology as a component of adaptation to a new environment. Three *Coffea* species, *C. arabica*,* C. canephora* (Robusta), and *C. liberica*, native to intertropical Africa have been introduced to New Caledonia. On this archipelago, a secondary contact zone has been characterized where these species coexist, persist, and hybridize spontaneously. We investigated the impact of environmental changes undergone by each species following its introduction in New Caledonia on flowering phenology and overcoming reproductive barriers between sister species. We developed species distribution models and compared both environmental envelopes and climatic niches between native and introduced hybrid zones. Flowering phenology was monitored in a population in the hybrid zone along with temperature and precipitation sequences recorded at a nearby weather station. The extent and nature of hybridization events were characterized using chloroplast and nuclear microsatellite markers. The three *Coffea* species encountered weak environmental suitability compared to their native ranges when introduced to New Caledonia, especially *C. arabica* and *C. canephora*. The niche of the New Caledonia hybrid zone was significantly different from all three species' native niches based on identity tests (*I* Similarity and *D* Schoener's Similarity Indexes). This area appeared to exhibit intermediate conditions between the native conditions of the three species for temperature‐related variables and divergent conditions for precipitation‐related ones. Flowering pattern in these *Coffea* species was shown to have a strong genetic component that determined the time between the triggering rain and anthesis (flower opening), specific to each species. However, a precipitation regime different from those in Africa was directly involved in generating partial flowering overlap between species and thus in allowing hybridization and interspecific gene flow. Interspecific hybrids accounted for 4% of the mature individuals in the sympatric population and occurred between each pair of species with various level of introgression. Adaptation to new environmental conditions following introduction of *Coffea* species to New Caledonia has resulted in a secondary contact between three related species, which would not have happened in their native ranges, leading to hybridization and gene flow.

## Introduction

Human activities, such as trade and cultivation, have increased the frequency of species introductions in a highly connected world, largely modifying species distribution patterns outside their native range and inducing significant range shifts (Parmesan 2006). Establishment of introduced species is usually modulated by both ecological and evolutionary processes depending on variations in biotic or abiotic conditions encountered in its new range. Among other phenomena, climate‐induced changes in life traits such as flowering phenology and new interactions with related species might promote interspecific hybridization.

Following the introduction of a species in a new territory, various scenarios may occur, ranging from extinction (Cooper et al. 1992) to adaptation and invasion (Broennimann et al. 2007). Comparing environmental conditions between native and new habitats could help to gain further insight into the dynamics following species displacements and to understand the fate of a species introduced in a new environment. Thus, the development of models characterizing the niche is a valuable tool for understanding and predicting how a species will be impacted by changing environmental conditions (Guisan and Thuiller 2005). There are two generally conflicting concepts regarding spatiotemporal species niche dynamics, that is, niche conservatism *versus* niche shift (Pearman et al. 2008). Although it has been suggested that climatic niche shifts are rare among plant invaders (Petitpierre et al. 2012), examples of niche shifts have mostly been demonstrated following the introduction of a species into a new environment (e.g., Broennimann et al. 2007; Mandle et al. 2010; Cornuault et al. 2015). Niche shift prevalence in some cases of introduction might explain situations of secondary contact between sister species (Benkman et al. 2008). But niche shift could also lead to divergence in physiological and life‐history traits (Allan and Pannell 2009).

One of the outcomes of the introduction of a species is a new ecological interaction with other species, both native and introduced (Abbott et al. 2003). Secondary contact between closely related species via translocation is especially prone to favor hybridization in plants and animals (Blair and Hufbauer 2010; Steeves et al. 2010). In plants, such translocation may quickly lead – in the absence of strong reproductive barriers – to hybridization and introgression with related native taxa, such as between species of *Ulmus* (Zalapa et al. 2010). According to Allendorf et al. (2001), there are three general patterns in hybridization between introduced and native species: (1) hybridization without introgression; (2) widespread introgression into the native species genome; and (3) complete admixture. To end up with complete admixture, the different phases of the process play over time and necessitate the adaptation and fertility of hybrids and advanced hybrids as gene exchange increases. Thus, the outcome of hybridization is highly diverse and depends on both the introduced species, other species involved in the hybridization and local environmental conditions.

The majority of studies on hybridization have been conducted on hybrids derived from crosses between an introduced and a native species. However, multiple related species may also be introduced to the same new environment, and hybridization between two introduced taxa may not be less uncommon than imagined. For example, Abbott (1992) calculated that of the 1264 nonnative plant taxa present in the British Isles in 1991, 21 (1.7%) were derived from hybridization between two introduced taxa. A rare documented example concerns radish (*Raphanus raphanistrum* and *R. sativus*) in which two nonnatives hybridized to form hybrid lineages with increased fitness (Campbell et al. 2006).

Moreover, few studies have considered secondary contacts between two related introduced species whose native ranges are geographically distinct (Cornuault et al. 2015). New secondary contact situations between sister species, whose flowering phenology evolved under different native climatic regimes, might alter reproductive isolation between them (Allendorf et al. 2001). Distinct flowering phenology among related species is an important prezygotic reproductive barrier maintaining species isolation by reducing cross‐pollination and gene flow. Under new climatic conditions, introduced species may display changes in flowering phenology (Cleland et al. 2007; Anderson et al. 2012), and altered differences in flowering phenologies could lead to hybridization (Lamont et al. 2003). Flowering time is a complex trait that is partially dependent on environmental factors (Petit et al. 1997), such as photoperiod (Imaizumi and Kay 2006), day length (Borchert et al. 2004), precipitation (Cowling et al. 2005), and temperature (Bustamante and Burquez 2008). These external signals influence a regulatory network so as to prevent a plant from flowering too early or too late in the season. The timing of the switch between vegetative and reproductive phases is crucial for efficient pollination and seed set for individuals and populations, so it is an important trait in plants and highly relevant for adaptation to climate change (Wolkovich et al. 2013). Evolution of locally adapted flowering times may occur over a relatively short period of time in plant species following introduction (Colautti and Barrett 2013). For example, in *Brassica rapa*, flowering phenology was shown to evolve rapidly in response to a multiyear drought (Franks et al. 2007). Thus, habitat‐induced flowering‐time shifts might promote new mating patterns and facilitate the evolution of local adaptation.

Coffee tree cultivation has a long history of introductions within multiple ecological contexts across the intertropical zone. Of the 125 *Coffea* species currently recognized (Davis et al. 2011), three are cultivated: *C. arabica*,* C. canephora* (Robusta) and, to a lesser extent, *C. liberica,* native to intertropical Africa. The characteristics and distributions of these three species are extremely diverse in terms of phenotypic and phenologic traits and niche occupation (Chevalier 1946; Davis et al. 2006). In particular, these species are seldom found sympatrically in African tropical forests, and the absence of flowering overlap, among other prezygotic barriers, is thought to generally prevent interspecific hybridization (Le Pierrès 1995). Only a few occurrences of spontaneous hybrids have been observed when these species are cultivated together, for example, the Timor hybrid on Timor Island (Cramer 1957; Bettencourt 1973) and in New Caledonia (Gomez et al. 2010a). Experimental hybridization between *Coffea* species has been reported and has produced relatively fertile hybrids, indicating the lack of complete postzygotic isolation barriers (Louarn 1992) and suggesting that prezygotic isolation, such as nonoverlapping flowering time, is important to maintain the integrity of species. Coffee tree flowering phenology is greatly controlled by climatic factors. The effects of light, soil moisture, and temperature on flowering success have been demonstrated in coffee agroforestry systems (Lin 2008). More specifically, coffee tree flowering generally occurs after a dry period releasing floral buds from dormancy, and the onset of occasional rains then triggers flowering and anthesis (Crisosto et al. 1992; Dinnan and Menzel 1994). The time between the triggering rain and flowering differs markedly between species and acts as the main reproductive barrier to gene flow in coffee species.

A unique situation has been characterized in the Sarraméa region of New Caledonia where three introduced coffee species were formerly cultivated. They presently coexist and hybridize spontaneously within abandoned plantations evolving only under the pressure of local environmental conditions (Gomez et al. 2010a,b). This situation of a secondary contact zone of introduced species with different evolutionary histories was investigated to understand the impact of environmental changes undergone by each species following its introduction in New Caledonia. We hypothesize that the changes in flowering phenology could have overcome the reproductive barriers between sister species.

The objectives of our study were to (1) investigate environmental changes undergone by each species following its introduction to New Caledonia through the comparison of both environmental envelopes and climatic niches between native and introduced hybrid zones; (2) evaluate the impact of climatic parameters on flowering phenology of sister species by monitoring the weather and their flowering patterns in the population of Sarraméa; and (3) evaluate the impact of flowering phenology on mating patterns in the three‐species population by genetic characterization of the extent and nature of hybridization events.

## Methods

### Study area and field sampling

Cultivated *Coffea* species, that is, *C. arabica*,* C. canephora* (Robusta), and to a lesser extent *C. liberica*, have been introduced from Africa into New Caledonia to support the local economy. First introduced in 1856 from La Réunion Island by Marist priests, coffee was widely cultivated during the colonial period, particularly by French “Feillet” colonists who settled in New Caledonia between 1894 and 1903. Native Melanesian tribes were also urged to cultivate coffee trees as a way for their communities to become integrated in a commercial economy (1931‐34) (Kohler and Pillon 1986; Leblic 2007). However, two pest introductions, that is, the coffee rust fungus (*Hemileia vastatrix*) in 1910 and the coffee berry borer (*Hypothenemus hampei*) in 1948, in addition to economic constraints, such as higher costs of labor, led to the decline of coffee cultivation. In particular, coffee plantations in the Sarraméa region were probably abandoned in the 1930s by the colonists' descendants. Trees from these plantations, previously managed in a “rustic coffee system” and then abandoned, have survived and hybridized for more than half a century without human intervention, leading to an unique hybrid zone (see Figure S1 in Supporting Information; Gomez et al. 2010a,b). *Coffea liberica* consists of two well‐differentiated subspecies, *C*. *liberica* ssp. *liberica* Hiern. and *C. liberica* ssp. *dewevrei* De Wild. and Dur. (N’ Diaye et al. 2005), but only *C. liberica* ssp. *liberica* has been introduced in Sarraméa (denoted hereafter as *C. liberica*) (Gomez et al. 2010a).

The reference three‐species (*C. arabica*,* C. canephora,* and *C. liberica*) population of Sarraméa (21°38.584′S, 165°51.733′E) was the focus of our genetic study. In this population, the circumference of individual trees was measured at 1 m height so as to reflect individual age, and only the 387 mature (i.e., flowering) individuals were genotyped, including some of the oldest trees (referred to as founders) and putative hybrids (assumed to be so on the basis of their morphological features). To ensure correct species assignments, we identified a representative subsample of specimens per species: two of *C. arabica*, 34 of *C. canephora* (representative of the diversity subgroups) (Gomez et al. 2009), and eight of *C. liberica* ssp. *liberica* (N’ Diaye et al. 2007). Both *C. liberica* and *C. canephora* are diploid species (2*x* = 2*n* = 22), while *C. arabica* is the only *Coffea* tetraploid species (allotetraploid; 2*n* = 4× = 44).

In this population, a subsample of 10 individuals of each species was also selected for monitoring flowering phenology.

### Genetic analyses

Nuclear genetic variation was assessed at twelve microsatellite loci (M259, Es18, Es62, Es70, Es82, Es90, M314, M387, M394, M495, M764, and M809). They were selected for being evenly distributed throughout the *Coffea* genome (Denoeud et al. 2014) and showing good amplification and high polymorphism information content (PIC) according to Poncet et al. (2006, 2007). PCR conditions and all information on the markers are given in MoccaDB (http://moccadb.mpl.ird.fr; Plechakova et al. 2009) and in the coffee genome hub (Dereeper et al. 2015).

Two chloroplastic microsatellite (cpSSRs) loci, with species‐specific alleles, were designed based on sequences published by Tesfaye et al. (2007). The first cpSSR, MS2 (alleles 225, 227, and 229 bp for *C. arabica*,* C. canephora*, and *C. liberica*, respectively), is located in the trnS‐trnG intergenic spacer and was amplified with the following primers: F:GCAAGGGGGTCTTCTTTAGTTT, R:GTCCACTCAGCCATCTCTCC. The second cpSSR, MS4 (alleles 180 and 182 bp for *C. liberica*/*C. arabica* and *C. canephora*, respectively), is located in the trnG intron. The sequences of primers used for amplification were as follows: F:TGGAAGGCTAGGGGTTATAG and R:TTCAGACAAAAGCTGACATAGA. Amplifications were carried out with a 60–55°C touchdown protocol according to Poncet et al. (2006). Maternal inheritance of chloroplastic DNA in *Coffea* (Lashermes et al. 1996) was confirmed on progenies (data not provided) and enabled us to monitor the crossing direction.

For both nuclear and chloroplastic microsatellites, amplified fragments were separated by capillary electrophoresis on an ABI 3130 Genetic Analyzer (Applied Biosystems, Waltham, MA), and fragment sizes were assessed using genemapper version 3.21 (Applied Biosystems) according to a size standard GS‐500 LIZ (Applied Biosystems). Automated allele calling was visually checked for each sample by two independent scorings (two persons). We finally retained 367 Sarraméa samples with good amplification and consistent amplification profiles. As *C. arabica* is an allotetraploid species that contains a considerable amount of fixed heterozygosity with maximum two alleles at each studied microsatellite, all samples including reference samples were coded as diploid.

Genetic polymorphism, including the observed number of alleles (Na), observed heterozygosity (*H*_O_), and expected heterozygosity (*H*_E_, or gene diversity), was assessed for each nuclear marker locus within the Sarraméa population using powermarker v3.25 software (Liu and Muse 2005). Principle coordinate analysis (PCA) was conducted using GenAlEx 6.5 (Peakall and Smouse 2012) based on the genetic distance (GD) estimated between individuals.

We analyzed the genetic relationship among Sarraméa individuals and hybrid detection using Structure v2.2.3 (Pritchard et al. 2000; Falush et al. 2003). This program performs comparably to other hybrid detection methods when used in a two‐species system (Burgarella et al. 2009), but it is the only software that allows assessing hybridization among three or more species. The most likely number of genetic clusters was estimated both with and without the African referent species, based on methods described in Evanno et al. (2005). Admixture degrees were estimated directly on the Sarraméa sample. Default settings were as follows: admixture model and allele frequencies correlated, 30,000 burn‐in steps, and 1,000,000 MCMC replicates. We assigned each individual to the inferred clusters using a threshold *q*_*i*_ > 0.80. In cases of admixed individuals, genotypes were considered as hybrids when the proportion of membership to each assigned group was 0.20 < *q*_*i*_ < 0.85. A 90% confidence interval of the *q*_*i*_ parameter was calculated for each individual.

### Niche analyses

The niche specificity of each species, both in native and in introduced contexts, was assessed based on niche models.

#### Data sources

Native range occurrence points for *Coffea arabica* (*n* = 42), *C. canephora* (*n* = 54), and *C. liberica* ssp. *liberica* (*n* = 12) were compiled from records of field collections in Africa (Meyer 1968; Berthaud and Guillaumet 1978; Berthaud et al. 1983; Anthony et al. 1985; Berthaud 1986; de Namur et al. 1987; Le Pierrès et al. 1989; Musoli et al. 2009).

The introduced hybrid zone is located in Sarraméa Valley, in the central part of the New Caledonian mountain range (Gomez et al. 2010a,b). We selected within this area 71 sites including two (without *C. liberica*) or three species, in order to characterize environmental conditions in the “introduced hybrid range” (Gomez et al. 2010b).

#### Environmental variables

Twenty environmental variables were considered as predictors in the three species distribution models: elevation and 19 bioclimatic variables described as biologically meaningful to define ecophysiological tolerances (Graham and Hijmans 2006). These variables were downloaded for the current conditions (years 1950 to 2000) from the WorldClim database (www.worldclim.org) at 30 arc‐second resolution (Hijmans et al. 2005) and resampled at 10 km resolution over intertropical Africa and New Caledonia. The absence of spatial correlation between variables was checked using ENMTools (Warren et al. 2010).

#### Models

The potential distribution (realized niche) of each of the three African species was modeled using maxent 3.3.3k software (Phillips et al. 2006). Based on a maximum entropy criterion, maxent combines presence‐only data and environmental layers to generate the distributions. Compared with other presence‐only models maxent shows good predictive accuracy (Elith et al. 2006) while not being sensitive to sample size effects (Wisz et al. 2008). We used the default maxent settings with a random test set of 25% of the input localities. We chose the “raw” output format for following comparison tests, as suggested by Warren et al. (2008). Jackknifing was performed to measure the variable importance, and model validation was conducted by calculating the receiver operating characteristic (ROC) and the area under the curve (AUC) (Phillips et al. 2006). We defined a 1000‐km buffer zone around all African and New Caledonian occurrence points, whatever the species, to obtain spatially comparable ecological niche models between species. This area encompassed relevant regions that have likely been accessible to the three species over their evolutionary history.

Ecological niche modeling was also implemented to characterize the introduced hybrid zone in New Caledonia based on hybrid population occurrence points.

#### Comparing the niches of native and introduced populations

The native distributions of the three African species were compared to each other and to that of the introduced hybrid zone in New Caledonia. Pairwise ecological niche overlap was quantified using Schoener's D (Schoener 1968) and the I statistic modified from the Hellinger distance (Warren et al. 2008). Both statistics vary between 0 and 1, with 0 indicating no niche overlap and 1 indicating identical niches. Differences were statistically tested in ENMTools (Warren et al. 2010). Background tests in ENMTools were also applied to assess whether the niches of two species are more or less similar based on differences in the environmental range (Warren et al. 2010). These tests were conducted by comparing the predicted niche of a focal species/hybrid zone with a set of pseudoniches modeled based on random sampling of the other species/hybrid zone geographic range according to Nakazato et al. (2010). We established an a priori definition of the range of each species (defining the background) as any grid cell within 50 km of known occurrences.

#### Comparing environmental envelopes between native and introduced ranges

Principal component analysis (PCA) was performed on the environmental conditions at occurrence points to compare the environmental envelope of the three African species in their native range using the “ade4” library in R software (Chessel et al. 2004). To visualize differences between the native and introduced hybrid range in New Caledonia, we included the New Caledonian occurrence points as additional passive elements within the previously implemented PCA.

### Flowering phenologies

We used several indices characterizing the patterns in flowering phenology, including frequency, duration, amplitude, synchrony, and regularity.

Flowering phenology was monitored on 11 individuals of *Coffea liberica*, 10 of *C. canephora*, and 10 of *C. arabica* of the Sarraméa population. Because of the heterogeneity of the hybrid types, involving each pair of species with various level of introgression (see below), not enough individual of each class could be monitored to infer trends with confidence. The individuals selected were randomly distributed in the population and representative of the size diversity sampled for each species. Observations were recorded during the 2008 flowering season from September to December. Several blossoming events may occur during a flowering season, which usually starts at the beginning of a rainy season (i.e., in September). Five events with different intensities were observed during the season.

**Figure 1:**
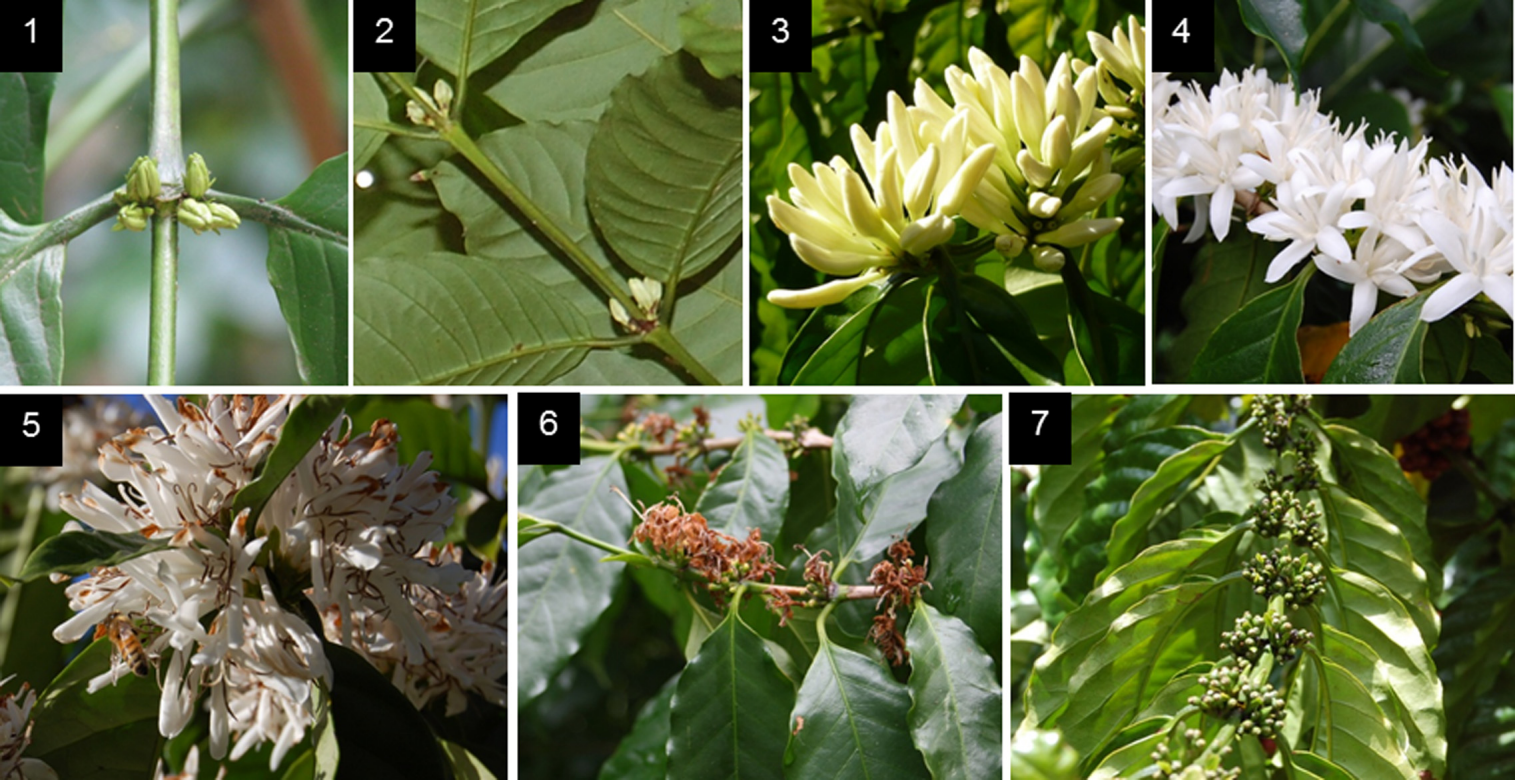
Flowering stages in coffee species. The seven distinct stages of coffee bud development along a flowering event were annotated as follows from bud initialization to fruit initialization: stage 1 = green cluster, 2 = white cluster, 3 = first white candle, 4 = anthesis, 5 = end of pollen viability, 6 = end of style receptivity, 7 = pinhead fruits.

An annotation grid was constructed according to the literature (Arcila‐Pulgarin et al. 2002) and known phenologies in Africa (Le Pierrès 1995), defining seven distinct stages of coffee bud development along a flowering event, as follows: stage 1 = green cluster, 2 = white cluster, 3 = first white candle, 4 = anthesis, 5 = end of pollen viability, 6 = end of style receptivity, 7 = pinhead fruits (Fig. 1).

Stage 4, which corresponds to male and female fertile stage, lasts only 1 day. Stage 5 is a female‐only fertile stage during which the style is still receptive but pollen is no longer viable. Observations were made daily during the blooming peaks (from stage 2 to stage 6) and weekly otherwise. Scoring was performed by consensus by two people. In order to maximize homogeneity, pictures of every observation were taken, allowing double‐checking and cross‐validation.

Flowering intensity classes were defined according to the number of flowers at anthesis (stage 4) observed on each tree: from 1 (only 1 to 3 flowers on the tree) to 4 (all branches blooming on the tree).

Under natural conditions, flower bud dormancy in *Coffea* is often broken by the first rains in the season following a dry period, sometimes associated with cool temperatures (DaMatta et al. 2007). To record temperature and precipitation sequences during the flowering season, a weather station (Davis Vantage Pro2, Davis Instruments, CIMA TECHNOLOGIE, Montanay, France) was installed next to the Sarraméa population.

## Results

### Genetic analysis

#### Admixture analyses of the Sarraméa population

Analysis of the 367 Sarraméa individuals revealed a total of 90 alleles for the 12 loci, giving an average of 7.5 alleles per locus, ranging from 3 to 11. The observed heterozygosity (*H*_O_) and gene diversity (*H*_E_) were also high, with an average of 0.48 and 0.70, respectively.

**Figure 2:**
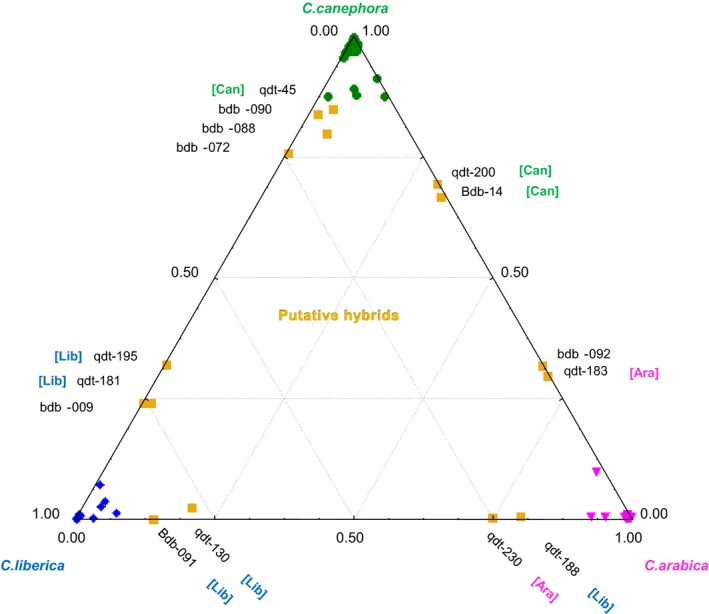
Genetic admixture analysis of the Sarraméa population (New Caledonia). Ternary plot of individual ancestry computed on the Sarraméa population (367 individuals) by structure with *K* = 3 and 12 microsatellite loci. Sample plotting at the apices have a proportion of membership close to qi = 1.0, that is, to pure species: *Coffea canephora* (green points), *C. liberica* (blue lozenges), and *C. arabica* (pink triangles). Hybrids and admixed individuals with at least 15% membership to a second species (yellow squares) are labeled with individual names and mother species chloroplastic origins are in brackets (*C. canephora*: [Can]; *C. liberica*: [Lib]; and *C. arabica*: [Ara]).

The analysis with STRUCTURE showed that three groups (*K* = 3) were genetically distinct based on SSR data (Fig. 2), both with and without African referent species. Individuals of each reference species, *C. canephora*,* C. arabica,* and *C. liberica,* clustered in one of the groups allowing the assignation of the Sarraméa individuals to each of the species. These distributions were congruent with principal coordinate analyses (see Figure S2). The admixture analysis was conducted using *K* = 3 on the Sarraméa individuals only. Three hundred and fifty‐two individuals were assigned to a species based on admixture coefficients above 0.85, while the remaining 15 (4%) were admixed among two species. Of the 352 individuals assigned to a “pure” species, 171 (48.6%) were *C. canephora*, 99 (28.1%) were *C. arabica*, and 82 (23.3%) were *C. liberica* (Fig. 2). Over the hybrid individuals, 12 were identified as admixed with over 0.20% ancestry from a second species, and the three other individuals with 15‐20%. These interspecific hybrids were represented with intermediate positions on the trispecific plot (Fig. 2). Admixture events occurred between each pair of species but, surprisingly, no F_1_ hybrid (an individual in which approximately half of the genome is from one species and half from another) was observed. Some individuals probably corresponded to a backcross hybrid of first generation (BC1), that is, having admixture values of 75% for one species and 25% for another, while others showed an advanced introgression level. The largest interspecific hybrid group was *C. liberica* × *C. canephora*, with seven individuals, compared to four *C. canephora* × *C. arabica* hybrids, and four *C. liberica* × *C. arabica* hybrids. The lack of detected F_1_ hybrids could be due to the fact that they might belong to the set of individuals that could not be properly amplified/genotyped, or that they were uprooted or cut when the plantation was still being managed, as noted by the many old coppiced *C. arabica* trees with double trunks.

Genotypes at the chloroplastic microsatellite loci (cpSSR) allowed us to determine the last cross direction. Backcrosses of second generation toward a specific species had the cpDNA of this species (figures between brackets in Fig. 2) in nine of 10 cases. This was higher than the expected frequency of 75%, and might indicate that the backcrosses were asymmetric, with F_1_ hybrids more frequently acting as fathers in backcrosses, independently of the female species.

### Influence on rainfall on the release, timing, and synchronization of anthesis

**Figure 3:**
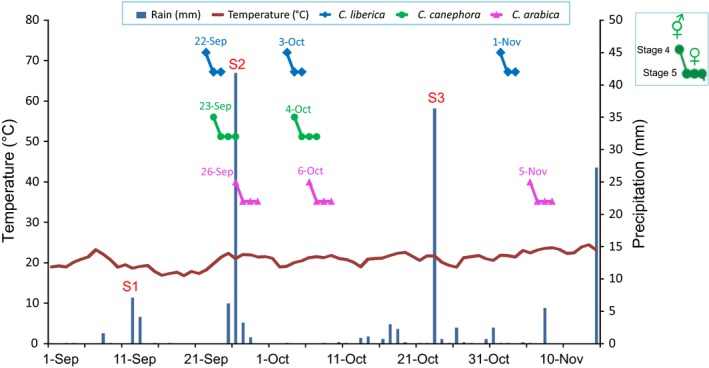
Time sequence of flowering patterns of the three *Coffea* species over a flowering season in Sarraméa. Flowering of *Coffea canephora*,* C. liberica* and *C. arabica* following rains during the blooming season at Sarraméa. Rainfall dates are indicated in S1–S3. Flowering stages 4 (anthesis, effective pollen and receptive style) and 5 (receptive style) are represented for the three flowering periods as validated on *c*. 10 individuals per species. Temperature and precipitation sequences were recorded by a nearby weather station (Davis Vantage Pro2, Davis Instruments).

Flowering phenology was surveyed on 10 individual plants of each species within the Sarraméa population, while temperature and precipitation sequences were recorded from a nearby weather station. The general time sequence of flowering events over the study flowering season is presented in Figure 3.

#### Species flowering events

Coffee floral buds in many environments usually develop for about 2 months after initiation and then become dormant for weeks or months. Anthesis is generally associated with a cycle of drought followed by rainfall (Schuch et al. 1992). During the studied year in Sarraméa, two major flowering events were recorded for *Coffea canephora* (23 September and 4 October), while three were recorded for *C. arabica* (26 September, 6 October, 5 November) and *C. liberica* (22 September, 3 October, and 1 November). Two supplementary events of low intensity were recorded for the latter.

Although each species underwent several successive flowerings during the season, their most intense bloomings were not at the same periods. The first flowering period was the main blossom for *C. canephora* (flowering pick), while the main blossom was at the second period and third one for *C. arabica* and *C. liberica*, respectively (see Figure S3).

#### Rain‐triggered flowering over the season

Each of the flowering events occurred after a substantial rain (over 10 mm in a single day) following a dry period (under 0.6 mm on average per day over a period of over 11 days) (Fig. 3, see Table S1). Temperature was relatively stable over the flowering season, with an average of 20.8°C. After breaking of dormancy, the triggering rain stimulated development of the flower buds to anthesis (stage 4) within 7 to 14 days, depending on the species. After each precipitation event, the three species flowered in the same order regardless of the date: *C. liberica* flowered first (7–10 days after rain), followed by *C. canephora*, 1 day later (8–11 days after rain), and then *C. arabica*, 3–4 days later (10–14 days after rain).

#### Overlapping flowering sequences

The three species had particularly brief and well‐synchronized flowering, whatever the date. All individuals of a species we observed flowered synchronously with stage 4 lasting a single day (anthesis, male and female fertile stage). On average over the study season, stage 5 (receptive style) lasted 2 days for *Coffea canephora*, 2.5 days for *C. liberica*, and 3 days for *C. arabica*.

Because of the brief flowering and the distinct flowering dates between species, only partial overlapping within a sequence was observed. Stage 4 (anthesis with both sexes fertile) never coincided between species, but stage 5 (receptive style) of one species may have overlapped with stage 4 of the following species. Likewise, when two flowering periods were close enough due to close triggering rains, flowering stage 5 of the last species (*C. arabica*) could have overlapped with stage 4 of the first species of next sequence (*C. liberica*). Interspecific pollen flow would thus be oriented according to the species flowering sequence, with a majority of hybrids showing one of the three interspecific combinations: *C. liberica* (♀) × *C. canephora* (♂), *C. canephora* (♀) × *C. arabica* (♂), and *C. arabica* (♀) × C. *liberica* (♂).

The presence of interspecific hybrids was indeed detected between each pair of species using both nuclear and chloroplastic markers but, because of the absence of identified F_1_ individuals in the population, we were not able to confirm the interspecific crossing direction.

### Niche comparisons

Environmental niche models (Phillips et al. 2006) were used to describe each species’ environmental requirements in their native ranges with the aim to assess environmental suitability for the species across their introduced zone in New Caledonia (Phillips et al. 2006).

#### Prediction and comparison of African native niches and environmental envelopes

Consistent with field observations and habitat descriptions of the species, we found substantial variation among the three *Coffea* species in terms of environmental requirement and predicted niche distribution (see Figure S4(A)). The *C. liberica* potential ecological niche was mostly located on coasts (mainly around the Gulf of Guinea), and the *C. canephora* putative ecological niche ranges from Guinea to Uganda, while that of *C. arabica* was mostly limited to East African highlands. Validation values were high for all models, with an AUC value of 0.99, 0.93, and 0.95 for *C. liberica*,* C. canephora*, and *C. arabica* models, respectively.

Randomization tests of niche identity confirmed that the members of each species pair were not ecologically equivalent (*P* < 0.01), regardless of the measure of similarity used (Schoener's D or I, Table 1). Although these differences could reflect evolutionary divergence of the species analyzed, they also might simply reflect the fact that populations of these species were exposed to different environmental backgrounds (see Figure S5). This hypothesis was however rejected by most of the background tests (Table 1), except for *C. liberica* and *C. canephora*, for which a small overlap was detected.

**Table 1:** Niche overlap among African native niches and the hybrid introduced zone in New Caledonia. Pairwise ecological niche overlap was quantified using Schoener's *D* and *I* statistics. Both statistics vary between 0 and 1, with 0 indicating no niche overlap and 1 indicating identical niches. The background test results are given for a species according to the background of the other species

Compared niches	Niche overlap index		Niche/Background	Background test	
	*I*	*D*		*I*	*D*
Native in Africa					
Can/Lib	0.62*	0.35	Can/Lib	0.87 ± 0.022	0.62 ± 0.04
	0.79 ± 0.06*	0.52 ± 0.014*	Lib/Can	0.46 ± 0.09	0.24 ± 0.06
Can/Ara	0.37*	0.14*	Can/Ara	0.87 ± 0.0022*	0.62 ± 0.039*
	0.90 ± 0.027*	0.66 ± 0.05*	Ara/Can	0.8 ± 0.03*	0.58 ± 0.041*
Lib/Ara	0.18*	0.06*	Lib/Ara	0.46 ± 0.0092*	0.25 ± 0.06*
	0.71 ± 0.04*	0.42 ± 0.04*	Ara/Lib	0.79 ± 0.031	0.58 ± 0.041*
Native/hybrid zone in NC					
Can/NC	0.07*	0.009*	Can/NC	0.91 ± 0.016*	0.72 ± 0.033*
	0.89 ± 0.32*	0.66 ± 0.054*	NC/Can	0.30 ± 0.016*	0.11 ± 0.01*
Lib/NC	0.14*	0.03*	Lib/NC	0.41 ± 0.19	0.21 ± 0.11
	0.64 ± 0.17*	0.38 ± 0.14*	NC/Lib	0.3 ± 0.16*	0.11 ± 0.16*
Ara/NC	0.016*	0.0028*	Ara/NC	0.8 ± 0.062*	0.56 ± 0.097*
	0.87 ± 0.07*	0.62 ± 0.11*	NC/Ara	0.30 ± 0.016*	0.11 ± 0.01

If marked with an asterisk *, the true calculated niche overlaps are outside the 95% confidence intervals and are therefore significant. Ara, Can, and Lib: native niches of *Coffea arabica*,* C. canephora*, and *C. liberica*, respectively; NC: hybrid zone niche in New Caledonia.

**Figure 4:**
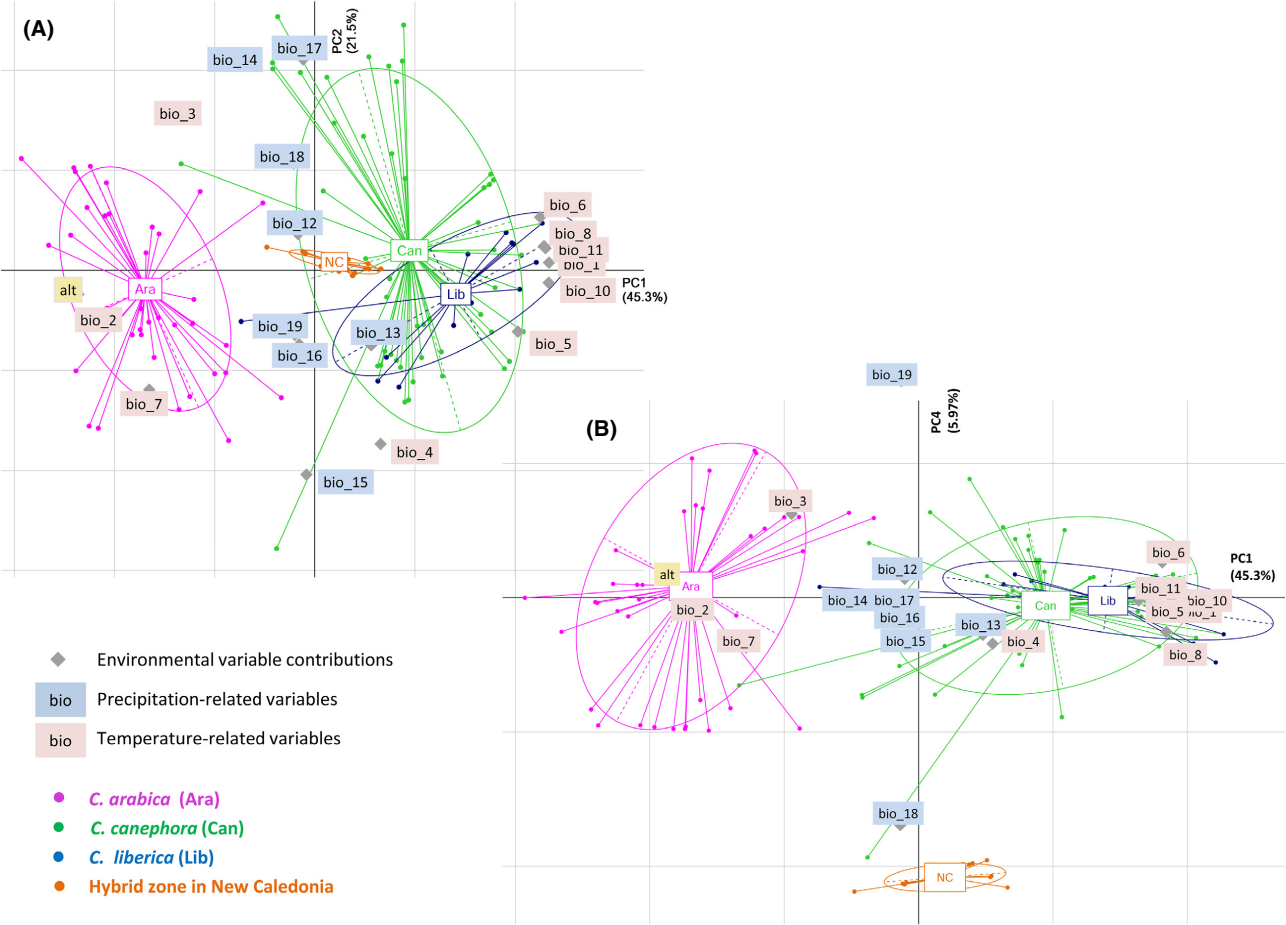
Principal component analysis (PCA) of the 19 bioclimatic variables and altitude in native and introduced (New Caledonia) records of *Coffea canephora*,* C. liberica*, and *C. arabica* occurrence. Distribution of the three species in the bioclimatic PCA space: (A) the *X*‐axis and *Y*‐axis show the first and the second principal components, accounting for 45.3% and 21.25% of the total variation, respectively (B) the *X*‐axis and *Y*‐axis show the first and the fourth principal components, accounting for 45.3% and 5.9% of the total variation, respectively. Native occurrence localities in Africa were treated as active variables while New Caledonian localities were treated as passive. Environmental variable contributions to both axes are represented on the same graph, while temperature‐related and precipitation‐related variables are shown in blue and pink, respectively. Ellipses representing environmental envelopes were automatically drawn.

The PCA based on environmental parameters also suggested the proximity of *C. canephora* and *C. liberica* ecological niches by overlapping their environmental envelopes and differentiating both of them from *C. arabica* along PCA1 (Fig. 4). Altitude and temperature‐related variables (e.g., Bio2, Bio10, Bio1, or Bio11) contributed mainly to this axis.

#### How suitable is the hybrid zone for the introduced species?

Niche projection for the three species over the introduced hybrid zone in New Caledonia revealed weak suitability of the environment for all species, although slightly better for *Coffea liberica* (see Figure S4(B)). The New Caledonia niche was significantly different from all three species’ native niches based on identity tests (Table 1). However, while no background effect was detected for *C. canephora* and *C. arabica* in the background tests, environmental differences weakly explained the ecological niche differences when the niche of *C. liberica* was considered in a New Caledonian background.

The passive projection of New Caledonian environmental conditions into the PCA based on environmental parameters (Fig. 4) revealed intermediary conditions between *C. canephora*–*C. liberica* and *C. arabica* on PCA1, determined mainly by the contribution of altitude and temperature‐related variables (e.g., Bio2, Bio10, Bio1, or Bio11). While PCA2 and PCA3 did not differentiate New Caledonia from the three native range conditions, a clear shift was illustrated along the PCA4 (Fig. 4). The main variables contributing to this axis were specific precipitation‐related variables, with in particular a higher level of precipitation in New Caledonia during the warmest quarter and a lower level during the coldest one (Bio18 and Bio19).

The niche in the introduced hybrid zone in New Caledonia appeared to provide intermediate conditions between the three species’ native conditions for temperature‐related variables and divergent conditions for precipitation‐related ones.

## Discussion

### Environmental shift mainly due to precipitation

Discrepancies noted when comparing the modeled range distributions of *Coffea arabica*,* C. canephora*, and *C. liberica* ssp. *liberica* in Africa suggested that major evolutionary and/or ecological processes have shaped the native niches of the three related species since their speciation (Yu et al. 2011). While *C. arabica* has a current natural distribution restricted to East African highland forests (Ethiopia, South Sudan, and Kenya), *C. liberica* ssp. *liberica* naturally grows in the lowland Congolese region (Côte d'Ivoire, Liberia) of West Africa and *C. canephora* has the widest distribution area, ranging from the Republic of Guinea to Uganda and from Cameroon to Angola, adapted to various forest types (Berthaud 1986; Davis et al. 2006). The three species are generally geographically and/or ecologically isolated from one another. Indeed, according to the results of our comparative distribution modeling, their realized ecological niches in Africa are differentiated, although this is less pronounced between *C. canephora* and *C. liberica* ssp. *liberica*. This is probably resulting from their partial natural coexistence in West Africa and their phylogenetic proximity (Maurin et al. 2007; Noirot et al. 2015), while a combination of current exposure to different environmental conditions and ongoing evolutionary niche diversification might explain part of their niche divergence.

The three *Coffea* species studied here were introduced in New Caledonia decades ago and encountered weak environmental suitability compared to their native ranges, especially *C. arabica* and *C. canephora*, but were nevertheless able to establish sympatrically in the Sarraméa region. In fact, microenvironments in the Sarraméa area have been also found to be favorable for the coexistence and hybridization of *Coffea* species (Gomez et al. 2010a,b). The present results suggest that species coexistence might be explained by an intermediate niche, mostly due to temperature‐related variables in Sarraméa that are intermediate as compared to the native conditions. On the contrary, a well‐marked niche shift in precipitation‐related variables likely impacted flowering phenologies and consequently weakened interspecific reproductive barrier.

### Flowering phenology tracks environmental conditions

Under different conditions from those experienced in the native range, selective processes can take place. The level of introduced genetic diversity and of phenotypic plasticity (Maron et al. 2004) would determine species adaptative abilities. Some species can evolve even over a short period by acclimatization, including shifts in gene expression, resource allocation, or morphology and physiology within the lifespan of an individual, especially for species that tend to be phenotypically plastic (Strayer et al. 2006). Flowering patterns over the year for the three *Coffea* species studied were shown to directly follow the timing and sequence of precipitation, tracing local climatic variations in Sarraméa, similar to what has been described in Africa (Le Pierrès 1995). The use of climatic cues to flower at a suitable time is critical to take advantage of environmental conditions that favor the production of fruits and seeds. It has been suggested that species that change their phenology in response to climate change are better at tracking suitable environmental conditions and therefore are more likely to persist when such changes occur (Cleland et al. 2012).

Diverse factors have been reported to contribute to flowering time regulation, particularly day length and vernalization pathways (e.g., Michaels 2009), but most studies have been conducted in temperate regions. Little is known about the way rain influences flowering time in tropical ecosystems. Unlike in other biomes such as temperate forests, phenology might be less sensitive to temperature and photoperiod, which tend to vary less as one gets closer to the equator, and more tuned to seasonal shifts in precipitation (Reich 1995; Sakai 2001). Within tropical forests, a broad spectrum of flowering patterns has been found (Sakai 2001), and observed variations in phenology may arise from phenotypic plasticity or from genetically based local adaptations or a combination thereof (e.g., Zalamea et al. 2011; Anderson et al. 2012).

Coffee flowering phenology has been characterized as highly dependent on climatic factors such as precipitation (Crisosto et al. 1992), photoperiod (Majerowicz and Söndahl 2005), and temperature (Lin 2008), and it is genetically controlled (Le Pierrès 1995). We obtained evidence of a genetic influence on flowering patterns, as seen in the way in which the three *Coffea* species studied flowered after a triggering rain. The time between this rain and the flowering date was well conserved, strictly species‐dependent, and followed a clear sequence in which *C. liberica* flowered first, followed by *C. canephora* and then *C. arabica*. This sequence has also been observed in Africa where blossoming occurs 5–6, 7, and 8 days after this rain for *C. liberica*,* C. canephora*, and *C. arabica*, respectively (Le Pierrès 1995). Moreover, all individuals of a species flowered synchronously. The genetic basis of the main flowering period was also recently suggested through interspecific controlled crosses between *C. canephora* and *C. pseudozanguebariae* (Akaffou et al. 2014). The fact that the flowering sequences were the same regardless of the environment – Africa or New Caledonia – and only depended on the intensity of the triggering rain suggests that *Coffea* species are able to track suitable environmental conditions, in particular precipitation, which might have facilitated the establishment of the species after their introduction.

Flowering patterns in *Coffea* thus appear to have a strong genetic component, causing the flowering times of each species to remain distinct. Moreover, flowering events over the study season showed distinct major peaks according to the species, which may suggest different period of attractiveness to pollinators. Partly asynchronous responses among species to key environmental factors would thus be expected to act as prezygotic barriers. However, we unexpectedly observed the contrary in the Sarraméa region. The clear climatic divergences regarding the precipitation regime between native and introduced ranges made these reproductive barriers less effective without weakening them.

### Rainfall regime favors interspecific hybridization

Indeed, bioclimatic variables in the Sarraméa region in New Caledonia are characterized by a less marked dry season (lower precipitation seasonality and higher precipitation during driest period and warmest quarter) than in the native ranges of the three African species studied. This appears to be congruent with the direct precipitation records from a weather station and a previous comparative study based on climogram analysis (Gomez et al. 2010a). The lack of a well‐marked dry season is considered to influence flower bud formation by creating different levels of induction (Crisosto et al. 1992) and favoring multiple flowering events during a season.

Moreover, because the triggering rains are closely distributed in time, the period between the successive flowering sequences is shortened and an overlap between species flowering is more likely to occur. In particular, stage 5 (when the style is receptive) of one species may overlap with stage 4 (anthesis, with both sexes fertile) of the next species, thus enabling interspecific gene flow. In fact, across the Sarraméa population, 4% of interspecific hybrids (15 individuals of 367 genotyped individuals) have been observed between each pair of species, independently of their ploidy level and with various levels of introgression. The presence of later generation hybrids resulting from backcrossing of F_1_ and later hybrid individuals provides clear evidence of hybrid fertility and successful gene flow. This zone is of exceptional significance as no similar phenomenon of spontaneous interspecific hybridization between *C. arabica* and *C. canephora* has been noted elsewhere in the world, except for a rare event observed in Timor leading to the so‐called Timor hybrid (Cramer 1957). Interspecific gene flows are currently well studied in natural long‐standing hybrid zones and provide a better understanding of the hybridization levels in relation to ecological factors (Lexer et al. 2005; Gow et al. 2006). However, understanding these factors in a case of introduction of several ecologically divergent species gives additional clue about which components of the climate are likely to be most important in generating the hybridization.

## Conclusion

Although the chances of adaptation and gene flow between the three *Coffea* species studied may have appeared a priori to have been relatively low in New Caledonia because of a lack of habitat suitability, the species coexist, persist, and hybridize spontaneously through the partial overlap of each species flowering sequence induced by the rainfall regime. In the future, shifts in precipitation are expected to occur worldwide concomitantly with increasing temperatures (Hartmann et al. 2013) and could generate new environmental situations affecting flowering patterns and thus reproductive barriers. Moreover, introgressive hybridization, by providing novel genetic combinations, might precede adaptation and evolutionary diversification and may result in a pathway to the rapid evolution of invasive forms (Ellstrand and Schierenbeck 2000). Although only 0.5% of the world's tree species are currently invasive outside of their natural range (Richardson and Rejmanek 2011), coffee trees may spread from planting sites and may become a threat like in the Indian Western Ghats (Joshi et al. 2009). Although coffee tree in the wild in New Caledonia does not seem to compete with native flora, it could be important to study the outcome of Sarraméa hybrids in terms of fitness and phenology, for example, in controlled conditions. This would help to predict and rank the invasiveness potential and future of the population, in terms of ecological and socioeconomic impacts by comparison with listed invasive species of New Caledonia (Meyer 2000).

## Conflict of Interest

None declared.

## Supporting information


**Figure S1**. Three‐species Sarraméa population.

**Figure S2**. Principal coordinate analysis of Sarraméa accessions and species referent samples based on their SSR polymorphism.

**Figure S3**. Bloom intensity over a flowering season in Sarraméa.

**Figure S4**. Species distribution predictions in their native and common introduced ranges.

**Figure S5**. Identity and background test for niche comparisons.

**Table S1**. Triggering showers and flowering of the three species.
