# Supplementary figures and images for: Shift in precipitation regime promotes interspecific hybridization of introduced Coffea species
Source: Ecol Evol. 2016 Apr 8;6(10):3240–55. doi: 10.1002/ece3.2055 (PMC4829533; doi:10.1002/ece3.2055)

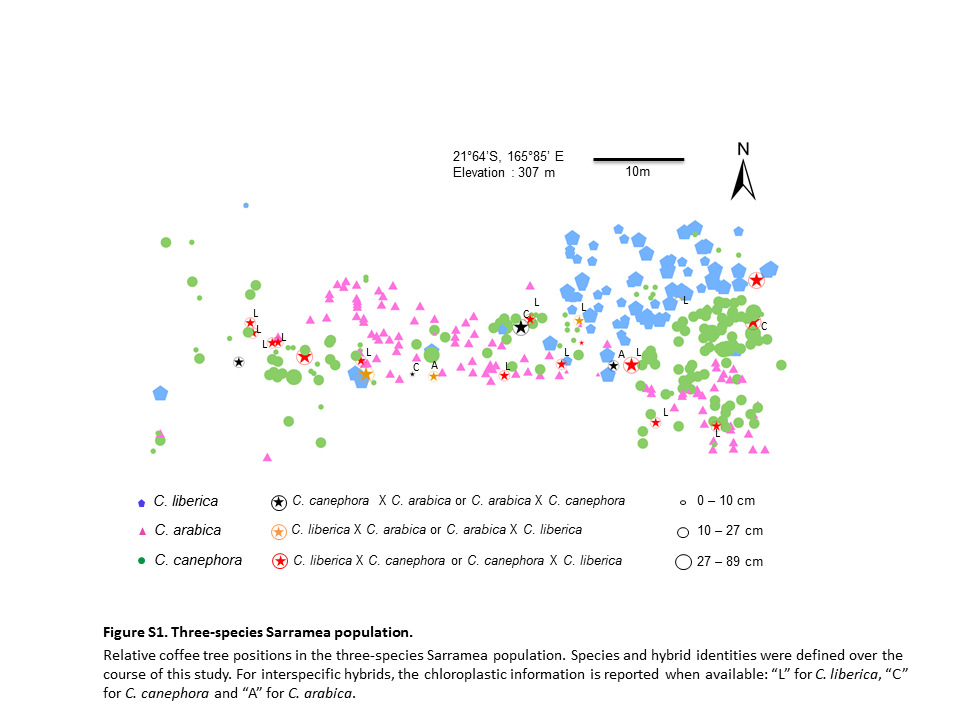

Supplement: Supplementary file 1 — Figure S1. Three‐species Sarraméa population. [file ECE3-6-3240-s001.tif]

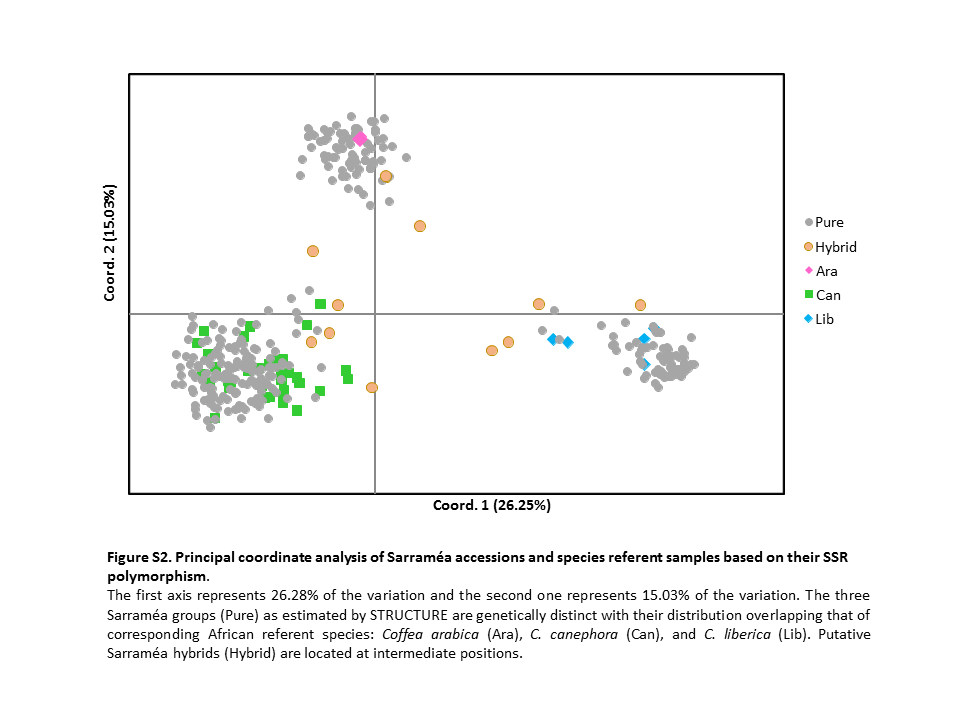

Supplement: Supplementary file 2 — Figure S2. Principal coordinate analysis of Sarraméa accessions and species referent samples based on their SSR polymorphism. [file ECE3-6-3240-s002.tif]

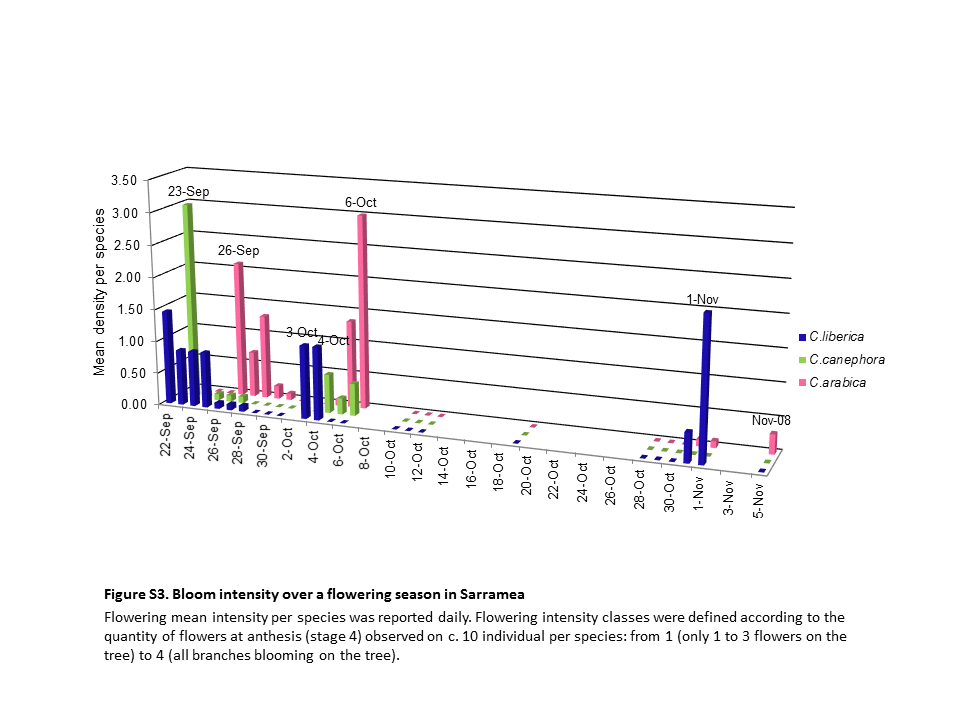

Supplement: Supplementary file 3 — Figure S3. Bloom intensity over a flowering season in Sarraméa. [file ECE3-6-3240-s003.tif]

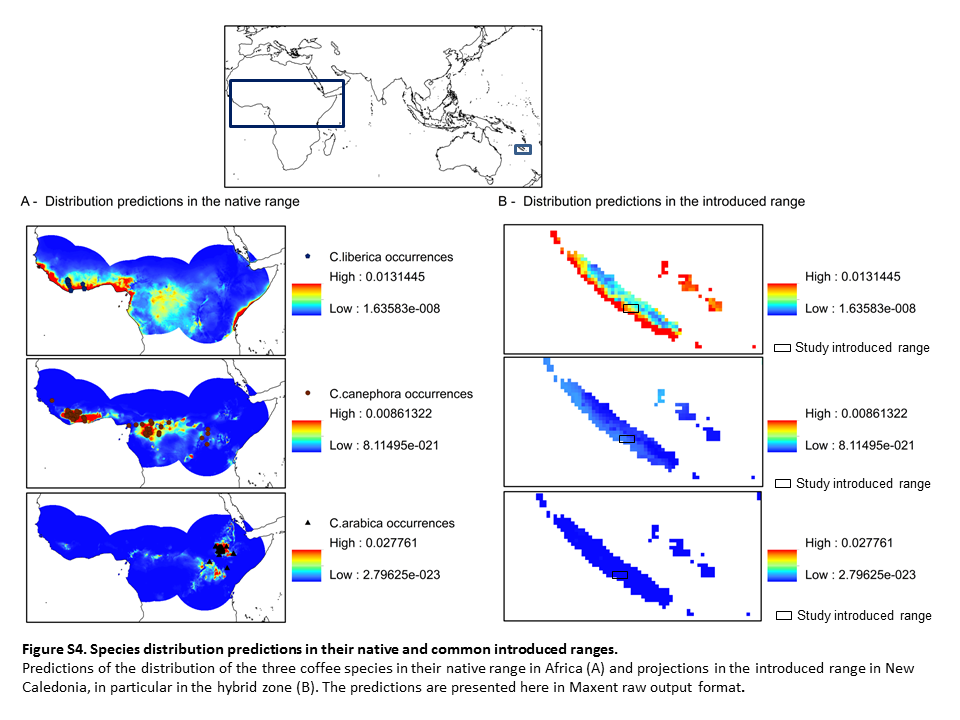

Supplement: Supplementary file 4 — Figure S4. Species distribution predictions in their native and common introduced ranges. [file ECE3-6-3240-s004.tif]
