# Supplementary material for: Shift in precipitation regime promotes interspecific hybridization of introduced Coffea species
Source: Ecol Evol. 2016 Apr 8;6(10):3240–55. doi: 10.1002/ece3.2055 (PMC4829533; doi:10.1002/ece3.2055)
Supplement: Supplementary file 5 — Figure S5. Identity and background test for niche comparisons. [file ECE3-6-3240-s005.pdf]

Figure S5. Identity and background test for niche comparisons.

Results of identity and background tests are given for every pair-wise comparison.

(1) Comparaison between each native niche: *C. arabica* / *C. canephora* (A), *C. arabica* / *C. liberica* (B) and *C. canephora* / *C. liberica* (C);

(2) comparaison between African native niches and the introduced niche in New Caledonia: *C. arabica* / New Caledonia (A), *C. liberica* / New Caledonia (B) and *C. canephora* / New Caledonia (C).

Niche similarity indexes are reported on the X-axis (I and D). Histogram bars represent the niche overlap values created in the replicates of the identity and the background test. Arrows indicate the results of an ENMtools niche overlap test representing the true calculated niche overlap. The background test results are given for the first species (according to the background of the second species) and the second species (according to the background of the first species). If marked with an asterisk *, the true calculated niche overlaps are outside the 95% confidence intervals and are therefore significant.

(1)

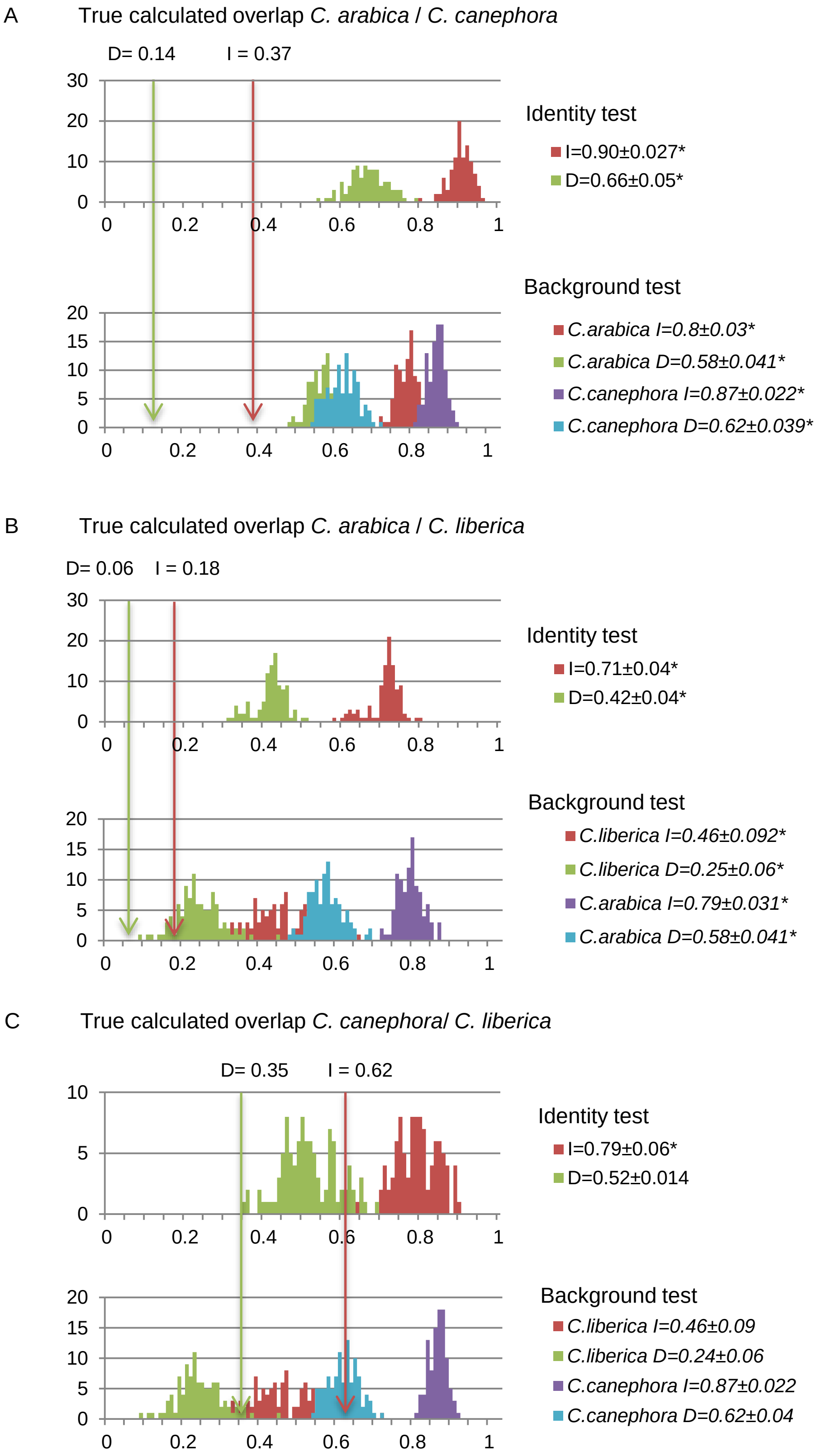

A
True calculated overlap C. arabica / C. canephora
D= 0.14
I = 0.37
Identity test
I=0.90±0.027*
D=0.66±0.05*
Background test
C.arabica I=0.8±0.03*
C.arabica D=0.58±0.041*
C.canephora I=0.87±0.022*
C.canephora D=0.62±0.039*
B
True calculated overlap C. arabica / C. liberica
D= 0.06
I = 0.18
Identity test
I=0.71±0.04*
D=0.42±0.04*
Background test
C.liberica I=0.46±0.092*
C.liberica D=0.25±0.06*
C.arabica I=0.79±0.031*
C.arabica D=0.58±0.041*
C
True calculated overlap C. canephora/ C. liberica
D= 0.35
I = 0.62
Identity test
I=0.79±0.06*
D=0.52±0.014
Background test
C.liberica I=0.46±0.09
C.liberica D=0.24±0.06
C.canephora I=0.87±0.022
C.canephora D=0.62±0.04

(2)

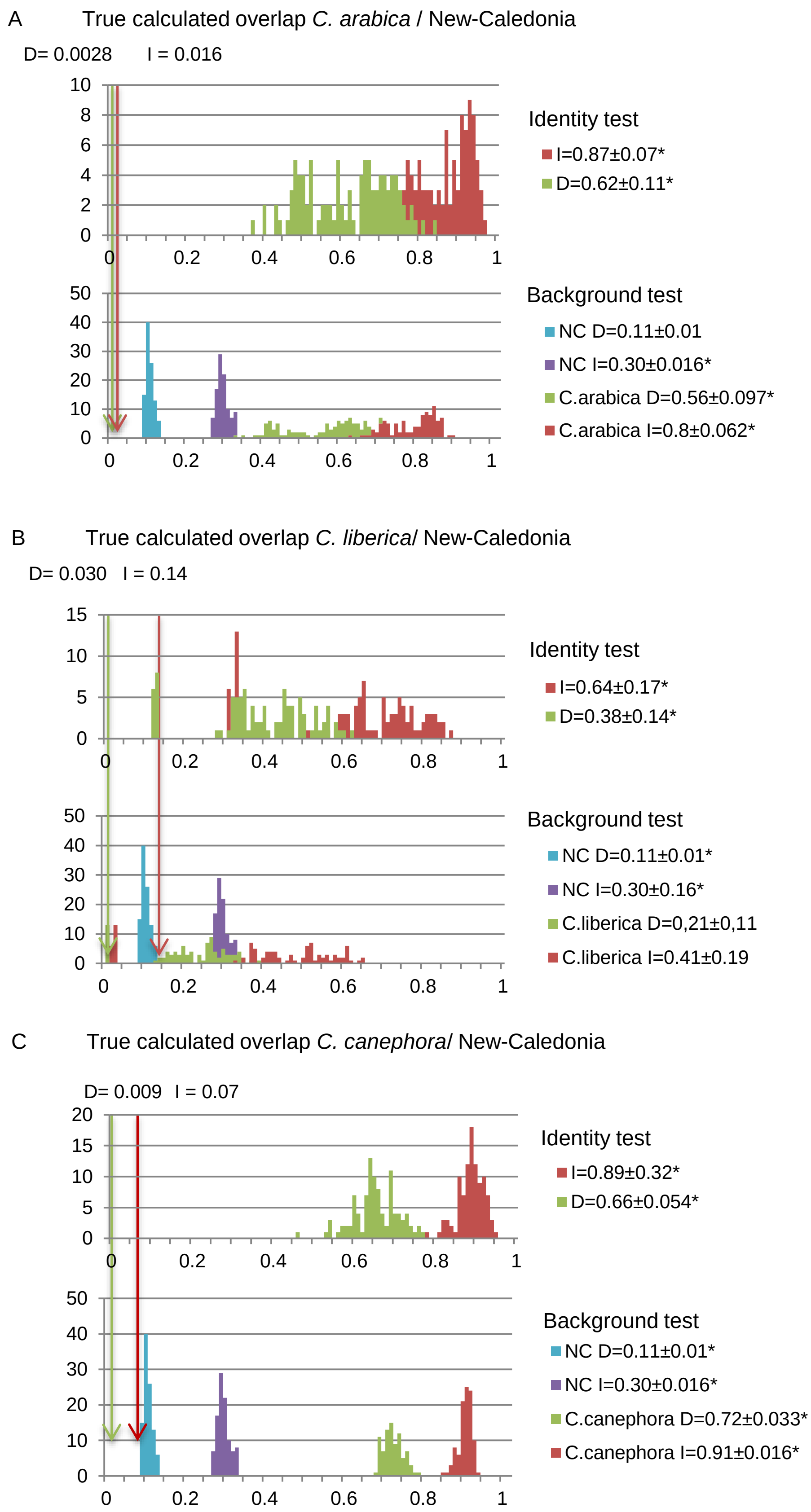
A
True calculated overlap C. arabica / New-Caledonia
D= 0.0028 I = 0.016
Identity test
I=0.87±0.07*
D=0.62±0.11*
Background test
NC D=0.11±0.01
NC I=0.30±0.016*
C.arabica D=0.56±0.097*
C.arabica I=0.8±0.062*
B
True calculated overlap C. liberica/ New-Caledonia
D= 0.030 I = 0.14
Identity test
I=0.64±0.17*
D=0.38±0.14*
Background test
NC D=0.11±0.01*
NC I=0.30±0.16*
C.liberica D=0,21±0,11
C.liberica I=0.41±0.19
C
True calculated overlap C. canephora/ New-Caledonia
D= 0.009 I = 0.07
Identity test
I=0.89±0.32*
D=0.66±0.054*
Background test
NC D=0.11±0.01*
NC I=0.30±0.016*
C.canephora D=0.72±0.033*
C.canephora I=0.91±0.016*
