# Supplementary material for: Shift in precipitation regime promotes interspecific hybridization of introduced Coffea species
Source: Ecol Evol. 2016 Apr 8;6(10):3240–55. doi: 10.1002/ece3.2055 (PMC4829533; doi:10.1002/ece3.2055)
Supplement: Supplementary file 6 — Table S1. Triggering showers and flowering of the three species. [file ECE3-6-3240-s006.doc]

**Table S1**: Triggering showers and flowering of the three species.

| Triggering showers | |  | Preceding dry period |  | Days after shower | | |
| --- | --- | --- | --- | --- | --- | --- | --- |
| (Days between showers) | Amount (mm) |  | Mean precipitation amount (mm/day) and length (days) |  | *C. liberica* | *C. canephora* | *C. arabica* |
| S1 | 11.4 mm |  | 0.3 mm -11 |  | 10 | 11 | 14 |
| S2 (+14) | 67 mm |  | 0.0 mm – 11 |  | 7 | 8 | 10 |
| S3 (+27) | 58 mm |  | 0.6 mm - 25 |  | 9 |  | 13 |
| S4 (+22) | 43.6 mm |  |  |  |  |  |  |
| S5 (+10) | 80.4 mm |  |  |  | 10 |  |  |
| S6 (+16) | 56 mm |  |  |  | 6 |  |  |
